# Constructing a dual-path motivation-regulation model of elderly sport consumption: a psychological perspective on behavioral drivers

**DOI:** 10.3389/fpsyg.2025.1628214

**Published:** 2025-06-24

**Authors:** Chong Zhou, Zehan Xu, Xiao Shi, Heng Liu, Yuanxin Chen

**Affiliations:** ^1^Physical Education and Sports College, Central China Normal University, Wuhan, China; ^2^Faculty of Science, The University of Sydney, Camperdown/Darlington Campus, Sydney, NSW, Australia

**Keywords:** elderly sports consumption, motivation-regulation framework, intrinsic motivation, external regulation, policy support

## Abstract

This paper presents a dual-path motivation-regulation framework for elderly sports consumption, aimed at comprehensively revealing how intrinsic motivation and external regulatory mechanisms jointly influence the behavioral decisions of older adults in sports consumption. The framework integrates Self-Determination Theory (SDT), Expectancy-Value Theory (EVT), and the Social Ecological Model (SEM), outlining two major paths: the motivation path and the regulation path. The motivation path highlights the internal psychological needs and emotional identification that drive elderly individuals in their sports consumption decisions. Key factors include consumer autonomy, perceived competence, emotional recognition, and motivational differentiation. The regulation path emphasizes the role of external factors such as family and social support, policy incentives, cultural identity, and product-market fit in shaping consumption behaviors. This paper also discusses the dynamic interaction and feedback loop between these two paths, showing that external regulatory factors can enhance intrinsic motivation, while intrinsic motivation can sustain consumption behavior despite weak external support. In conclusion, the paper provides policy and practical recommendations, focusing on strengthening intrinsic motivation through autonomy and confidence-building, enhancing external regulation through supportive family, institutional, and cultural frameworks, and adapting the market to better meet elderly consumers’ needs. This study offers a theoretical foundation for promoting elderly sports consumption and provides actionable insights for policymakers, social organizations, and commercial entities to foster long-term engagement and development in elderly sports consumption.

## 1 Introduction

The accelerating global trend of population aging has heightened scholarly and policy attention toward the health status, well-being, and overall quality of life of the elderly. According to recent estimates, the number of people aged 60 years and older is projected to increase from 1.1 billion in 2020 to 1.4 billion by 2030, and to exceed 2.1 billion by 2050, placing significant demands on public health and social systems (World Health Organization, 2019; United Nations, Department of Economic and Social Affairs, 2022). In response to these demographic shifts, the concept of healthy aging has gained prominence as a guiding framework for ensuring not only physical longevity but also enhanced quality of life in later years. Within this framework, the quality of life and well-being of older adults are influenced by a complex interplay of interrelated factors. Empirical studies have demonstrated that engagement in social and daily living activities significantly reduces feelings of loneliness among the elderly (Sau et al., 2015). Moreover, participation in various forms of leisure activities, such as visiting friends, playing games, and running errands, serves as an indirect pathway that encourages more active involvement in physical activities (Parra-Rizo et al., 2022). In institutional settings, interventions that integrate high-intensity resistance training and walking with personalized social engagement have been shown to significantly enhance the daily functioning of older residents (Lorenz et al., 2012). Furthermore, the specific types of physical activities pursued by older adults are associated with distinct dimensions of their quality of life, as different modalities of exercise may have varying impacts on psychological well-being and social integration (Sanchís-Soler et al., 2025). The UN Decade of Healthy Ageing 2021–2030 explicitly incorporates the promotion of physical activity as a key component of its strategic framework for fostering healthy ageing (World Health Organization, 2022). In the context of population aging, older adults can significantly enhance their quality of life and well-being, and promote healthy aging through active participation in social networks, physical activities, and daily life interactions.

Among the multifaceted strategies for promoting healthy aging, sports consumption has emerged as a critical domain, as it contributes to maintaining physical functionality, reducing chronic disease risks, improving life satisfaction, and fostering social integration (Dause and Kirby, 2019; Paár et al., 2021; Gao et al., 2022; Kim et al., 2024). In this study, sports consumption is conceptualized as a dual construct: behavioral participation in physical activity and commercial engagement with sports-related goods and services. While the commercial aspect is relatively straightforward, the behavioral aspect also constitutes a form of consumption. Participation in physical activity though non-commercial requires significant investment of time, bodily effort, attention, and emotional involvement, all of which reflect the consumption of personal and social resources. This interpretation aligns with broader conceptualizations of experiential consumption, particularly within leisure and health economics literature, where sports activities are viewed as hedonic, identity-expressive, and health-oriented experiences (Koo and Lee, 2013; Jenkin et al., 2018; Straker et al., 2021).

Elderly sports consumption takes diverse forms. It goes beyond mere physical activity and also includes broader lifestyle practices. From a physiological perspective, certain health-related behaviors, such as the consumption of wild-caught fish, may pose heightened risks to older adults due to their increased susceptibility to environmental toxins accumulation (Kouali et al., 2022). This underscores the necessity for age-specific guidance on health-oriented sports consumption. Concurrently, physical exercise is widely recognized as a pivotal factor in promoting functional longevity. Empirical studies suggest that regular physical activity not only aids in maintaining hormonal balance and overall physical health (Fiore et al., 1989), but also enhances psychosocial well-being by alleviating loneliness and fostering emotional engagement (Kim et al., 2011). Moreover, collective sports practices, such as group workouts, community-based fitness programs, and fan-based social networks, create meaningful social spaces that offer emotional support and a sense of belonging for older individuals.

In addition to active engagement in sports, older adults increasingly consume health-related products, including dietary supplements, wearable fitness trackers, and therapeutic wellness equipment. These behaviors symbolize the extension of a sports-oriented lifestyle and serve practical functions in health maintenance and disease prevention. Particularly in the context of public health emergencies such as the COVID-19 pandemic, the demand for products that enhance immunity and resilience has grown markedly (Miranti, 2020), reflecting a broader shift toward holistic, proactive, and self-regulated health consciousness among aging populations. Importantly, the motivations underlying elderly sports consumption are multidimensional and complex. They range from physical health maintenance and nostalgic emotional activation to psychological gratification, social interaction, and the pursuit of self-actualization (Malchrowicz-Mośko and Chlebosz, 2019). Despite a growing body of empirical evidence, the academic community still lacks a comprehensive psychological framework capable of systematically explaining the dynamic interaction between internal motivational forces and external regulatory conditions. However, many existing studies continue to conceptualize elderly consumers as passive, homogeneous, or overly rational actors, thereby neglecting the complex motivational structures and environmental contingencies that influence their behavior (Moss and Wilson, 2018; Rejeski and Fanning, 2019; Sung and Yoon, 2024).

In response, this study proposes the Dual-Path Motivation-Regulation Model for elderly sports consumption. To address the psychological mechanisms underlying elderly sport consumption behavior, this study proposes a Dual-Path Motivation–Regulation Model that integrates three complementary theoretical perspectives: Self-Determination Theory (SDT), Expectancy-Value Theory (EVT), and the Social Ecological Model (SEM). SDT explains how the satisfaction of basic psychological needs (autonomy, competence, and relatedness) stimulates intrinsic motivation and supports behavioral sustainability. EVT adds a cognitive-evaluative dimension, highlighting how individuals’ expectations of success and subjective valuation of outcomes shape consumption decisions. SEM broadens the analysis by embedding individual behavior within multi-level contextual systems, including interpersonal relationships, community resources, and policy environments. Based on these integrated insights, the proposed model delineates two interrelated pathways: an intrinsic motivation pathway, driven by psychological need fulfillment and value internalization, and an external regulation pathway, shaped by social, institutional, and environmental influences. By bridging internal drives with external conditions, the model offers a comprehensive and policy-relevant framework for understanding and promoting elderly sport consumption in the context of healthy aging. This model integrates core intrinsic motivational factors, such as autonomy, competence, and value identification, with extrinsic regulatory influences stemming from family structure, institutional arrangements, cultural norms, and market mechanisms. By explicating the interaction between these two pathways, the model offers a more nuanced and explanatory perspective on the initiation, maintenance, and cessation of sports consumption behaviors in later life.

## 2 Theoretical findings: a dual-path psychological mechanism of elderly sports consumption behavior

With the advancement of the healthy aging agenda, understanding the psychological mechanisms behind elderly sports consumption behavior is not only of theoretical importance but also foundational for upgrading sports consumption and delivering more precise services. This paper proposes a “Dual-Path Motivation–Regulation Model,” constructed based on three complementary classical theories: Self-Determination Theory (SDT), Expectancy-Value Theory (EVT), and the Social Ecological Model (SEM). This model integrates both individual motivations and contextual constraints to explain elderly sports consumption from two pathways: the intrinsic motivation pathway and the external regulation pathway. It reveals the logic behind the formation of consumption intention, the mechanisms sustaining consumption behavior, and the limiting factors, thus providing theoretical support for promoting the shift “from passive exercise to active consumption.”

### 2.1 Self-determination theory: activating intrinsic motivation for sports consumption

Self-determination theory (SDT), developed by Ryan and Deci (2000), emphasizes the continuum of human motivation, from autonomous, intrinsically driven behavior to controlled, externally regulated actions. At the core of SDT are three psychological needs: autonomy, competence, and relatedness. When these needs are satisfied, individuals experience enhanced well-being and long-term behavioral persistence (Flannery, 2017). SDT has been applied in a range of domains, from occupational health to academic achievement. For example, physicians driven by autonomous motivation report better occupational health outcomes, underscoring the role of intrinsic aspirations in well-being. In educational contexts, motivation profiles derived from SDT have been linked to student success and engagement (Matos Sousa et al., 2024). Within sports, intrinsically motivated athletes tend to perform better and experience higher levels of eudaimonic well-being (Kouali et al., 2022). Teaching strategies that support autonomy, competence, and relatedness have also been found to increase student motivation in physical education (Koka and Hagger, 2010).

SDT posits that individual motivation lies on a continuum from external regulation to autonomous self-regulation, driven by the satisfaction of three basic psychological needs: autonomy, competence, and relatedness. SDT is highly applicable in the context of elderly sports consumption. Many elderly consumers choose sports products and services that align with their financial capacity and health needs precisely because they satisfy the following:

***Autonomy**: Having the ability to afford the costs independently;*

***Competence**: Gaining health benefits and self-confidence from using the products or services;*

***Relatedness**: Engaging in emotional bonding and social interaction by discussing the products or services with others.*

However, aging-related factors, such as declining income, deteriorating physical function, emotional fluctuation, and social isolation, can hinder the fulfillment of these psychological needs, disrupt the internalization of motivation, and affect behavioral sustainability. Therefore, SDT not only explains the psychological basis of elderly sports consumption, but also suggests intervention paths to stimulate intrinsic motivation, such as enhancing the sense of choice, competence, and social identity through family support and public welfare policies, thereby fostering sustainable consumption momentum.

### 2.2 Expectancy-value theory: revealing the cognitive evaluation process of consumption decisions

Expectancy-value theory (EVT), articulated by Eccles (1984) and Wigfield and Eccles (2000), posits that motivation is a function of individuals’ expectancy of success and the subjective value they assign to a task. These two components jointly determine behavioral choices, intensity of effort, and long-term commitment. EVT has been extensively applied in educational research, showing that academic motivation is shaped by beliefs in personal competence and the perceived importance, enjoyment, and usefulness of the learning task (Doménech-Betoret et al., 2017). In second-language learning (Wang and Xue, 2022), physical activity among individuals with impairments (Kirk et al., 2021) and cognitive rehabilitation (Choi et al., 2010), EVT has consistently demonstrated its relevance in explaining engagement through the dual lens of expectancy and value. In the digital learning era, EVT has provided insights into learner persistence in MOOCs, emphasizing the roles of self-efficacy and course relevance (Lee and Song, 2022). Moreover, its integration with SDT in studies of international education has shown that intrinsic aspirations significantly predict behavioral intention (Yue and Lu, 2022).

EVT argues that an individual’s behavioral choices depend on their expectations of success and the subjective value assigned to the task. This theory expands the cognitive explanatory dimension of motivation and is particularly useful in understanding the rational evaluation mechanisms of elderly sports consumers. In practice, older adults typically assess the following:

“Can this product or service help improve my health?” (Expectancy)

“Is this product or service worth my time and money?” (Self-efficacy)

“What health or emotional benefits will it bring?” (Value)

“Will it pose safety risks or extra costs?” (Cost)

EVT’s strength lies in its emphasis on the multidimensional nature of value, including:

***Intrinsic value**: the enjoyment and satisfaction derived from the activity itself;*

***Utility value**: the functional benefits to health or self-care ability;*

***Attainment value**: the sense of achievement gained;*

***Opportunity cost**: the time, energy, emotional burden, and financial expenditure involved.*

For example, some elderly individuals may avoid personal fitness coaching due to high perceived cost (e.g., fear of injury) but are willing to pay for socially engaging activities with friends. EVT thus provides essential cognitive logic to explain “why buy,” “willing to buy,” and “what to buy,” and can support the development of differentiated supply strategies based on psychological value positioning.

### 2.3 Social ecological model: expanding the contextual regulation dimension of sports consumption

While self-determination theory (SDT) and expectancy-value theory (EVT) focus on individual-level determinants, the social ecological model (SEM; Bronfenbrenner, 1979; McLeroy et al., 1988) provides a broader, multilevel framework that situates behavior within interacting individual, interpersonal, community, societal, and policy contexts. This approach is especially valuable for analyzing complex issues such as health behaviors and social participation. For example, in addressing micronutrient access, SEM highlights the interplay of personal habits, social networks, and macro-level policies, guiding integrated public health strategies (Stavitz, 2024). In childhood nutrition, SEM supports nursing interventions that consider parenting, caregiving settings, and policy environments (Oudat and Okour, 2025). Dietitians use SEM to identify barriers to weight management, including low socioeconomic status and limited health literacy and emphasize the role of structured programs and supportive environments (Aboueid et al., 2019). The model also informs equity-focused pharmacy education by mapping curricular and community-level responses to racism (Nonyel et al., 2021), and it helps unpack the complex decision-making dynamics in kidney transplantation among Black and Latino patients (Wong et al., 2022). In the context of the opioid crisis, SEM reveals multilevel risk factors across personal, relational, and systemic domains, emphasizing the need for coordinated responses (Jalali et al., 2020).

SEM advocates examining behavior as socially embedded within a multi-level structure from individual, relational, and community to policy levels. It emphasizes that behaviors are shaped and maintained through the interaction of individual motivation, social support, environmental opportunities, and institutional arrangements. In the context of elderly sports consumption, SEM effectively reveals which external factors facilitate or hinder consumption behavior. For instance:

***Microsystems**: whether family members, neighbors, and caregivers support the consumption decision;*

***Mesosystem**: the accessibility of community fitness facilities, senior universities, and public activities that ensure basic conditions for consumption;*

***Macrosystem**: elderly-friendly policies, sports consumption subsidies, and health-promoting cultural environments.*

Even when elderly individuals have sufficient motivation, they may abandon consumption due to inconvenient transportation, lack of appropriate sports products, or participation barriers (e.g., digital divide that hinders online shopping). Conversely, robust external support systems can compensate for limited intrinsic motivation and serve as catalysts for behavior activation and persistence. SEM thus injects a “consumption niche” perspective into the model, highlighting the crucial role of systematic support in elderly consumption behavior.

### 2.4 Theoretical integration: constructing the dual-path motivation–regulation model

By integrating SDT, EVT, and SEM, this paper’s proposed “Dual-Path Motivation-Regulation Model” offers a systematic explanation of elderly sports consumption behavior. The model includes two core pathways: Intrinsic Motivation Pathway: Centered on SDT, it explains how satisfying basic psychological needs drives sustained consumption; External Regulation Pathway: Co-constructed by EVT and SEM, it reveals how elderly individuals make consumption decisions based on value expectations and contextual support.

These two paths are not mutually exclusive but dynamically interactive and mutually reinforcing. Individual motivation can be activated by social support or suppressed by adverse environments; sustained consumption behavior relies not only on intrinsic interest but also on external resources and policy support.

The innovation of this model lies in conceptualizing sports consumption as a psychological-contextual dual-driven process, combining micro-level motivations with macro-level structures. It emphasizes both the agency of older adults and their vulnerable realities. This contributes not only to theoretical advancement in elderly consumption research but also provides a conceptual tool for designing sports services, building incentive mechanisms, and optimizing policy systems.

## 3 Model construction: the dual-path motivation–regulation framework

This chapter, based on Self-Determination Theory (SDT), Expectancy-Value Theory (EVT), and the Social Ecological Model (SEM), proposes a dual-path motivation–regulation model for elderly sports consumption. The model aims to comprehensively reveal the internal motivational forces and external regulatory mechanisms that jointly influence the behavioral decisions of older adults in the context of sports consumption. Through two intertwined yet distinct paths, the model illustrates how motivational and regulatory factors synergistically shape the psychological and behavioral dimensions of elderly sports consumption.

The dual-path framework is designed to reflect the interactive relationship and co-development of intrinsic motivation and external regulation in elderly sports consumption, constituting a complex mechanism underlying their consumption behaviors. This framework consists of two major paths:

***Motivation Path (Intrinsic Drive)**: This path is primarily driven by internal psychological needs, including autonomy, competence, relatedness, value internalization, and emotional resonance. It reflects the individual’s inherent motivation to engage in sport consumption based on personal interest, identity, and enjoyment.*

***Regulation Path (External Drive)**: This path is shaped by external regulatory factors such as family and social support, cultural and social norms, institutional incentives, and the policy environment. These elements influence participation through perceived task value, expected outcomes, environmental facilitation, and contextual reinforcement.*

These two paths work together to shape the psychological motivation and behavioral performance of the elderly in sports consumption.

### 3.1 Motivation path: the intrinsic drive of elderly sports consumption

The motivation path centers on the intrinsic psychological needs and emotional identification that shape elderly individuals’ decisions regarding sports consumption. Grounded in Self-Determination Theory (SDT) and Expectancy-Value Theory (EVT), this pathway encompasses four critical dimensions.

Consumer autonomy reflects older adults’ desire for self-directed choice in selecting sports-related products and services that align with their health goals, preferences, and financial considerations (Wertenbroch et al., 2020). This autonomy supports their psychological need for self-governance and control, particularly in tailoring consumption decisions, such as selecting low-intensity exercise equipment suited to their physical condition, which enhances their engagement and satisfaction in the consumption experience.

Perceived competence plays a vital role in shaping sports consumption behaviors (Guo and Huang, 2025). It refers to the sense of effectiveness and control elderly consumers feel when using sports products to manage their health. When they engage with user-friendly fitness equipment or health-monitoring devices specifically designed for seniors, observable improvements in physical condition or well-being often ensue. These positive outcomes reinforce their sense of efficacy, further motivating sustained participation in sports-related consumption.

Emotional and identity-based recognition highlights the role of affective experiences and self-concept in driving motivation (Inoue et al., 2020). For many elderly individuals, sports consumption is not merely utilitarian but deeply tied to emotional gratification and identity affirmation. Purchasing high-quality fitness products or participating in age-targeted sports programs can elicit feelings of enjoyment, pride, and social belonging. These experiences foster a sense of being active, capable, and socially integrated, thereby strengthening the internalized motivation to continue engaging in such behaviors over time.

Motivational differentiation underscores the stratified nature of elderly individuals’ sports consumption motivations, which range from intrinsic drives to various forms of extrinsic regulation (Sheehan et al., 2018). At one end of the spectrum, intrinsic motivation is characterized by a genuine interest in sports activities, often stemming from the pursuit of physical well-being, emotional enjoyment, and overall quality-of-life enhancement. Moving outward, identified regulation reflects conscious alignment with personal health values, whereby older adults engage in sports consumption because they recognize its importance to their long-term well-being. Introjected regulation, by contrast, is shaped by internalized social expectations, such as the desire to meet family approval or to fulfill perceived duties to remain healthy for the sake of others. At the outermost layer, external regulation arises from direct external pressures, including government-led health campaigns, policy incentives, or community-organized programs that encourage participation. Recognizing these diverse motivational layers provides a more nuanced understanding of the underlying psychological drivers that govern elderly sports consumption behavior.

### 3.2 Regulation path: the external drive of elderly sports consumption

The regulation path emphasizes the influence of external environmental factors on the sports consumption behaviors of the elderly, highlighting how these factors can either facilitate or hinder their participation. Among the most critical elements is family and social support (Zhou et al., 2023). Family members, particularly spouses and children, as well as broader social networks, serve as powerful regulatory agents. Their encouragement and involvement can significantly bolster older adults’ confidence and willingness to participate in sports-related activities. Engaging in joint physical activities with family members or receiving motivation from peers fosters a socially supportive atmosphere that lowers psychological barriers and strengthens commitment to sustained consumption.

Institutional and policy support also plays a pivotal role in shaping elderly sports consumption (Misuk et al., 2018). Public institutions, health management systems, and government-driven initiatives such as fitness subsidies for seniors, the development of age-friendly sports facilities, and targeted health promotion programs collectively help to remove financial and infrastructural constraints. These institutional mechanisms reduce participation thresholds and provide structural incentives that stimulate older adults’ motivation and ability to consume sports services and products.

Cultural and societal norms further influence the regulatory landscape (Jun and Choi, 2024). In certain socio-cultural contexts, aging is often associated with passivity or physical decline, and sports are seen as the domain of the young. Such stereotypes may suppress older individuals’ motivation to participate in fitness-related consumption. Reconstructing these narratives through public discourse and media representation to promote the value of active aging can contribute to more inclusive social norms. As positive portrayals of elderly participation in sports become more prevalent, older adults may feel more socially validated and motivated to engage.

Finally, the fit between market offerings and the specific needs of older consumers is essential for enabling sustained engagement (Levinger et al., 2021a; Kang et al., 2025). Products such as health monitoring wearables, ergonomically designed low-impact fitness equipment, and age-adapted exercise programs must align with the physical capabilities, safety concerns, and usage habits of the elderly. When sports products and services are tailored effectively, they not only improve accessibility and usability but also foster a sense of respect, recognition, and brand loyalty among older consumers. This alignment between supply and demand is key to ensuring the inclusiveness and sustainability of elderly sports consumption.

### 3.3 Dynamic interaction and feedback loop

The intrinsic motivation and external regulation paths do not function independently; rather, they are dynamically intertwined and mutually reinforcing. External regulatory factors, such as family support, social recognition, and policy incentives, can strengthen intrinsic motivation by enhancing individuals’ sense of value and perceived competence (Fransen et al., 2018; Crawford et al., 2020). In turn, strong intrinsic motivations, such as emotional enjoyment, autonomy, and a sense of identity, empower older adults to maintain engagement in sports consumption, even in the face of environmental or structural barriers.

Over time, these two mechanisms form a self-reinforcing feedback loop. On the one hand, supportive external conditions can amplify intrinsic drivers, fostering sustained consumption behavior. On the other hand, intrinsic motivation can buffer the negative impact of insufficient external support, helping older adults persist in their consumption patterns. This bidirectional interaction underpins the psychological resilience and long-term continuity of elderly sports consumption.

### 3.4 Moderating variables

Individual differences significantly influence both motivational and regulatory pathways. Factors such as socioeconomic status, health condition, cultural background, and education level impact sports consumption behavior. For instance, older adults with lower income may be unable to afford premium sports products, while cultural norms may restrict participation (Tsuji et al., 2021). Identifying and addressing these differences is key to developing targeted incentive measures for diverse elderly populations.

### 3.5 Final model synthesis: a visual schema of the dual-path psychological mechanism

In light of the preceding theoretical elaborations grounded in Self-Determination Theory (SDT), Expectancy-Value Theory (EVT), and the Social Ecological Model (SEM), this section synthesizes the dual-path motivation-regulation framework into a systematic visual representation. The proposed model is not merely a structural abstraction, but a theoretically informed schema that explicates the psychological underpinnings and contextual determinants of elderly sports consumption behavior.

**Figure 1:**
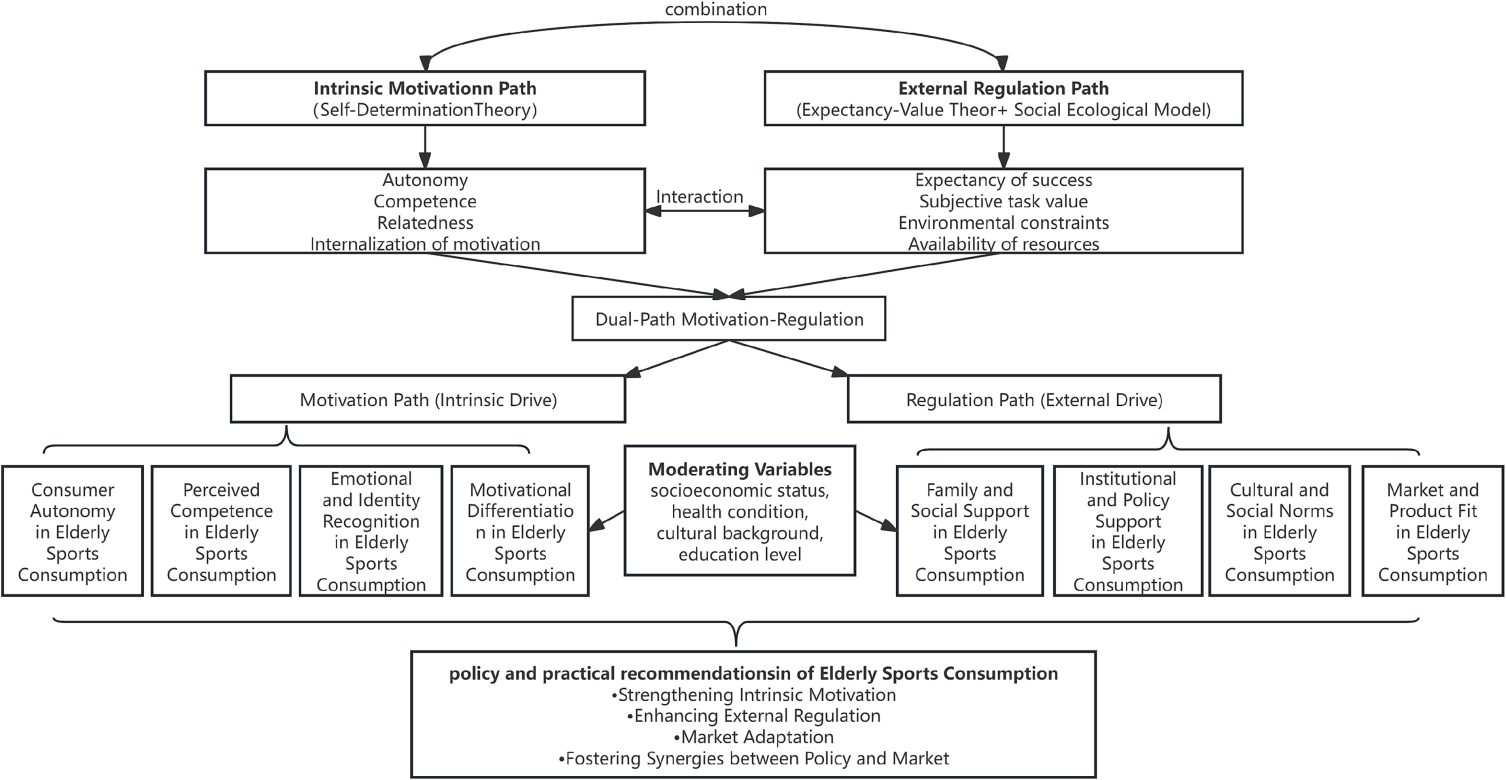
Dual-path psychological mechanism of elderly sports consumption behavior.

By delineating the motivation path and the regulation path the model captures the bidirectional causality and dynamic interdependence between internal psychological dispositions and external regulatory forces. The integration of moderating variables such as socioeconomic status, health condition, and cultural background ensures that the model accommodates individual heterogeneity, thereby enhancing its explanatory robustness and applicability across diverse population segments. The final diagram (Figure 1) thus serves as a comprehensive and operationalizable framework, laying the foundation for subsequent empirical validation and policy-oriented applications in the domain of aging and active consumption.

## 4 Recommendations for promoting elderly sports consumption through the dual-pathway model

Building upon the previously constructed dual-path motivation-regulation model, this section presents a set of policy and practical recommendations designed to holistically promote elderly participation in sports consumption and support its sustained development. These recommendations are organized along two interrelated dimensions: the intrinsic motivation pathway and the external regulation pathway. By aligning psychological incentives with structural supports, the proposed strategies aim to provide actionable guidance for policymakers, community organizations, and commercial stakeholders to jointly create an enabling environment for active and meaningful elderly sports engagement.

### 4.1 Strengthening intrinsic motivation to promote sports consumption

To enhance intrinsic motivation, three strategic directions are particularly essential. To begin with, expanding personalized consumption options is crucial. Both governments and enterprises should offer a broader array of sports products and services tailored to the preferences and physical conditions of older adults. Customizable offerings in areas such as fitness equipment, sportswear, and activity types can empower the elderly to make autonomous choices aligned with their health goals and lifestyle, thereby heightening their sense of agency and enjoyment (Levinger et al., 2021b). For instance, Low-intensity fitness programs customized to the physiological characteristics of older adults, together with ergonomically designed exercise equipment, are increasingly recognized as key instruments for promoting both health outcomes and enriched consumption experiences in the aging population (Evans, 1999). These interventions not only enhance cardiovascular function, muscle strength, and balance, but also alleviate anxiety, improve mental well-being, and elevate overall life satisfaction. As a salient example, outdoor fitness equipment (OFE), embedded within natural exercise environments, offers accessible and user-friendly physical activity spaces, thereby increasing older adults’ participation willingness and lowering entry barriers to physical engagement. Such adaptive fitness solutions not only foster sustained exercise behaviors but also serve as vital catalysts for stimulating sports consumption motivation and expenditure (Marcos-Pardo et al., 2023; Kersu and Kosgeroglu, 2025), making them integral to the expansion of an inclusive and health-oriented elderly consumption system.

In addition, fostering a sense of self-efficacy plays a vital role. Public health institutions and community organizations should develop training and education programs focused on health management and physical literacy for older adults (Garcia et al., 2014). Such programs, emphasizing basic exercise techniques, safety guidance, and health improvement plans suitable for this demographic, can help the elderly perceive tangible benefits from physical activity, thereby reinforcing their internal drive to consume sports-related goods and services.

Equally important is nurturing emotional identification and social identity through sports participation. Elderly sports consumption is not only a path to better health but also a channel for fulfilling emotional and social needs (Stenner et al., 2020). Policies that promote inclusive cultural atmospheres, encouraging participation in community fitness programs, sports clubs, and group activities, can strengthen a positive image of vitality and enhance social belonging. Moreover, media campaigns highlighting inspiring stories of active aging can contribute to shaping a confident and healthy elderly identity.

### 4.2 Enhancing external regulation to support elderly sports consumption

On the regulatory front, constructing robust support systems is essential for sustained engagement. Strengthening familial and social support is a foundational step. Policies that promote intergenerational participation, such as incentives for family-based fitness activities or bundled household exercise packages, can significantly increase older adults’ motivation. A systematic review identified various factors that influence the participation of older adults in such programs, emphasizing the importance of understanding these factors to develop successful intervention strategies (Zhou et al., 2024). In addition, community-driven initiatives that foster peer interaction and shared experiences can reinforce participation through social reinforcement mechanisms. Furthermore, broader systemic policy support is needed to lower access barriers. Governments should introduce targeted incentives for elderly sports participation, including subsidies, discounts for public sports facilities, and free health management services (Ribeiro et al., 2015). These measures not only alleviate financial burdens but also signal institutional endorsement of active aging. Infrastructure improvements, such as age-friendly facility designs, are equally vital to ensure safe and convenient access for elderly users.

At the cultural level, transforming societal narratives around aging is imperative. Stereotypes that frame the elderly as unfit for physical activity must be dismantled (Brothers and Diehl, 2017; Fernández-Ballesteros et al., 2020). Through public education campaigns and the promotion of positive role models, society can cultivate a cultural ethos that embraces senior participation in sports. This shift not only fosters recognition of the elderly as capable and active but also enhances their cultural legitimacy as sports consumers.

### 4.3 Tailoring products and services to meet the sports consumption needs of the elderly

To meet the growing and diverse needs of elderly consumers, the market must respond with adaptive strategies. Developing suitable products and customized services should be a top priority. Businesses are encouraged to design and offer specialized equipment, low-intensity fitness programs, and user-friendly wearable devices tailored to the elderly population. These innovations can improve accessibility, safety, and enjoyment, thereby increasing the willingness to participate and consume. Simultaneously, the integration of health management with sports consumption presents a promising avenue. With technological advancements, companies can introduce smart devices, such as health-monitoring wearables that allow older adults to track and manage their health in real-time, such as computer-based cognitive training (CCT) and socially assistive robots (SAR), which have shown promise in promoting healthy aging and improving cognitive functions among older adults. These technologies can help ease daily activities and enhance the quality of life for the elderly (Alnajjar et al., 2019). Furthermore, the nutritional needs of elderly consumers are often overlooked, despite their importance for public health and the food industry. The heterogeneity of the elderly market necessitates targeted segmentation strategies to effectively address these needs. The food industry can better cater to the nutritional preferences and requirements of older adults. This approach not only benefits the consumers but also provides valuable insights for product design and communication strategies (van der Zanden et al., 2014). Such innovations bridge the gap between daily life and fitness engagement, aligning sports consumption with broader wellness goals.

### 4.4 Coordinating policy and market efforts to stimulate sports consumption

Effective promotion of elderly sports consumption also depends on coordinated efforts between policy and market actors. A dynamic interplay between the two is essential: while policy interventions can create favorable market conditions through incentives, the market, in turn, can fulfill public goals by offering targeted products and services that meet elderly consumers’ demands (Saltzman et al., 2015). For instance, tax benefits or subsidies for businesses that develop age-friendly fitness products can encourage innovation and responsiveness. Government subsidies have been shown to promote corporate innovation by providing financial relief and encouraging firms to overcome financial constraints, which can otherwise suppress innovation efforts (Li et al., 2021). In addition to direct financial incentives, creating a supportive regulatory environment can further encourage businesses to innovate. By leveraging these strategies, governments can create an environment where businesses are motivated to develop products that promote healthy aging (Anderson et al., 2011). Additionally, cross-sector collaboration must be strengthened. Governmental bodies across domains, such as health, civil affairs, sports, and social security, should work together to form an integrated support system. One study highlights the importance of cross-sector collaboration in public health systems, emphasizing the need for partnerships between health care, social services, and other sectors to address social determinants of health (Hamer and Mays, 2020). Through interdepartmental coordination and resource pooling, a more comprehensive service ecosystem can be established to address economic, psychological, and social barriers faced by the elderly in sports consumption.

## 5 Conclusion

This study introduces a Dual-Path Motivation-Regulation Model to explain the psychological underpinnings of sport consumption behavior among the elderly. By integrating theories of self-determination, expectancy-value, and ecological influence, the model highlights the dynamic interplay between internal motivations, such as autonomy, competence, self-identity, and external regulations, including social support, cultural norms, and institutional interventions. Rather than treating elderly sport consumption as a purely health-driven or economically rational behavior, this framework underscores its multidimensional nature: a psychosocial practice shaped by both individual agency and structural context. To distinguish this model from purely academic frameworks, we emphasize its potential for practical application. Specifically, the model can serve as a theoretical foundation for designing personalized interventions that enhance motivation through autonomy support, value alignment, and social connectedness. It also provides guidance for policymakers to develop inclusive and elderly-friendly sports environments ranging from accessible infrastructure and service design to incentive policies and community engagement programs. By aligning psychological mechanisms with environmental supports, the model offers a strategic tool to promote physical activity, emotional well-being, and social integration in aging societies. Future research should empirically validate the model across different socio-cultural contexts to refine its generalizability and optimize its utility in public health and social policy.
